# Reframing early warning for Nipah virus in Bangladesh: A shift from detection to anticipation

**DOI:** 10.1371/journal.pntd.0014525

**Published:** 2026-07-16

**Authors:** Md. Shakil Ahmed

**Affiliations:** Department of Biochemistry and Molecular Biology, Faculty of Science, University of Rajshahi, Rajshahi, Bangladesh; University of Calgary, CANADA

## Abstract

Nipah virus continues to pose a recurrent zoonotic threat in Bangladesh. The sporadic spillover events are associated with high case fatality and limited opportunities for clinical intervention. Despite nearly two decades of surveillance response, early warning systems remain reactive, relying heavily on laboratory confirmation and case accumulation. This Perspective argues that early warning has been conceptually misframed as outbreak detection, rather than risk anticipation. Drawing on the epidemiological characteristics of Nipah virus and Bangladesh’s current surveillance architecture, this study highlights key structural constraints delaying signal recognition. These include community-level blind spots, diagnostic uncertainty, and fragmented cross-sectoral surveillance. This article also outlines priority shifts that emphasize anticipatory risk assessment, operational One Health integration, context-sensitive alert thresholds, and a direct linkage between early signals and preparedness actions. Reframing early warning in this way is essential to improve readiness for the Nipah virus and other rare, high-impact zoonotic threats.

## 1. Introduction

Nipah virus remains one of the most lethal zoonotic threats in Bangladesh. It causes recurrent spillover events with a high case fatality rate of approximately 71%, despite nearly two decades of outbreak surveillance and response [1]. While the number of cases remains low, each event carries disproportionate public health consequences. This is due to rapid clinical deterioration, limited treatment options, and the potential for human-to-human transmission [1].

Despite sustained attention, early warning for the Nipah virus continues to depend on laboratory-confirmed outbreaks, with limited capacity for early identification of spillover risk [1]. This misalignment reflects a broader conceptual challenge: early warning has been treated as an extension of outbreak confirmation, rather than as a mechanism for risk anticipation and early decision-making under uncertainty. Evidence from other rare zoonotic diseases shows a similar pattern, where surveillance systems designed primarily for outbreak detection fail to enable timely preparedness [2].

This Perspective argues that strengthening early warning for the Nipah virus in Bangladesh requires a fundamental shift in surveillance logic. Rather than asking how to detect the Nipah virus earlier, the more relevant question is how to recognize heightened risk before clinical detection thresholds are crossed. By examining structural gaps in current systems and outlining priority shifts, this article highlights how preparedness systems can be reoriented toward proactive response rather than delayed action.

## 2. Rethinking “early warning” for Nipah virus

Early warning in public health refers to the systematic detection, reporting, and interpretation of signals that may indicate increased disease risk before an outbreak is fully established or widely confirmed [3]. In the context of the Nipah virus, such systems should not be equated with confirmation of early outbreaks. The epidemiology of Nipah—characterized by sporadic spillover, low incidence, and high case fatality rate (40–75%)—renders traditional surveillance thresholds poorly suited for timely alerts [1,4]. Waiting for multiple confirmed cases undermines the very purpose of early warning for rare but high-impact pathogens.

An effective framework of early warning for the Nipah virus must prioritize sensitivity to atypical signals over numerical increases in case counts. Such signals include clusters of unusual encephalitis, severe illness with fever and epidemiological links, or cases occurring during seasons of known high risk [5]. Importantly, early warning must extend beyond clinical detection to incorporate recognition of contextual risk, including ecological, behavioral, and seasonal factors associated with spillover in Bangladesh [6]. These factors are closely linked to the ecology of reservoir hosts, particularly *Pteropus* bats, whose feeding behavior, habitat overlap with humans, and seasonal activity patterns shape the timing and location of spillover risk. However, surveillance of these populations remains limited in practice [7].

**Fig 1:**
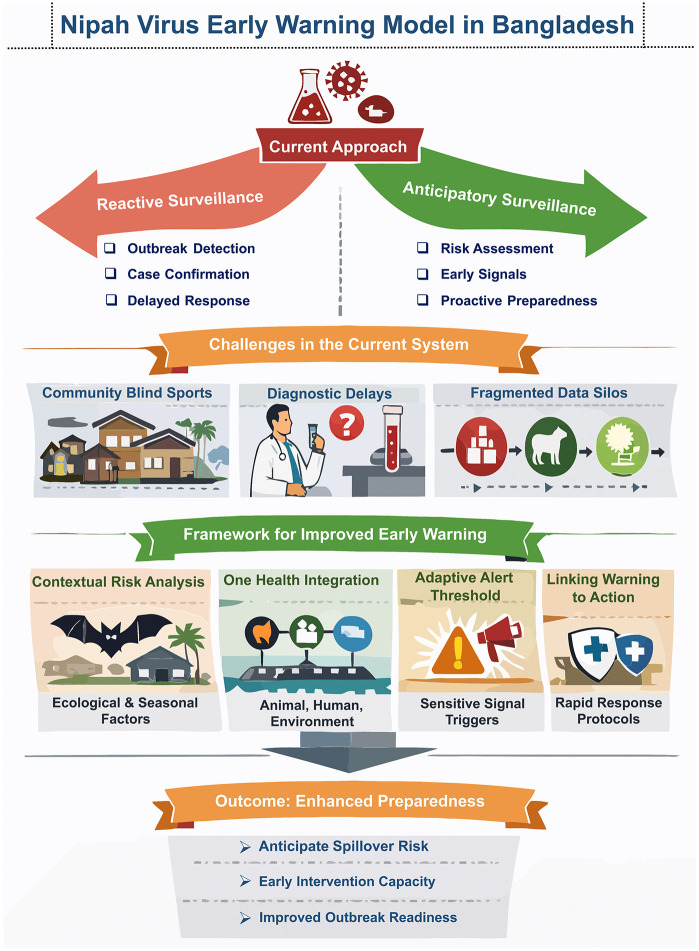
Conceptual framework for reframing early warning for Nipah virus in Bangladesh. The figure contrasts conventional reactive surveillance—based on outbreak detection and laboratory confirmation—with an anticipatory early-warning approach focused on spillover risk recognition. It highlights key structural constraints within the conventional system, such as community-level blind spots, diagnostic delays, and fragmented cross-sectoral surveillance. The proposed framework emphasizes contextual risk analysis, operational One Health integration, adaptive alert thresholds, and a direct link between early-warning signals and preparedness actions. This shift aims to enhance readiness for rare, high-impact zoonotic threats. The figure was prepared in Adobe Illustrator, version 2024.

This distinction mirrors broader public health debates that differentiate early warning for preparedness from surveillance designed primarily for burden estimation or outbreak confirmation. Early warning for the Nipah virus is therefore best understood as a dynamic process of risk-based monitoring, interpretation of early signals, and timely decision-making under uncertainty (Fig 1). Without this reframing, surveillance systems will continue to identify Nipah virus only after opportunities for prevention and containment have narrowed.

## 3. Structural constraints that delay early warning

Despite sustained attention to the Nipah virus in Bangladesh, existing early-warning mechanisms remain constrained by structural limitations that delay signal detection and risk escalation [8]. These gaps are not attributable to an absence of surveillance activities, but rather to how, when, and where signals are generated and interpreted (Fig 1).

### 3.1 Community-level blind spots

Current surveillance systems rely heavily on facility-based reporting. During 2006–2021, long-term surveillance data from Bangladesh indicate that Nipah detection has historically relied on hospital-based encephalitis surveillance, which has identified approximately 68% of reported Nipah cases [1]. While this system has been effective for case confirmation, it inherently captures cases at a later stage of disease progression rather than at the point of spillover, limiting its sensitivity to early community-level signals.

Early signals emerging from communities—where spillover events typically originate—are frequently overlooked. A recent community-based study in Bangladesh found that approximately 68% of individuals with Nipah-like illness did not seek care at a Nipah-surveillance hospital, while 24% first consulted informal providers [9]. This indicates that a substantial proportion of early cases may remain outside formal surveillance pathways. Consequently, small clusters of unusual illness may not be detected until severe clinical presentation occurs [8].

Community health workers could serve as critical sentinels for early warning by actively tracking local spike patterns, identifying initial clusters of severe encephalitis or respiratory distress, and serving as a direct link between rural communities and central epidemiological agencies [10]. Yet, in practice, they face structural barriers to doing so, including the absence of standardized reporting triggers for unusual clusters, limited integration into formal surveillance pipelines, and a lack of feedback mechanisms that would incentivize proactive reporting. This failure limits the system’s ability to recognize early risk amplification.

Public awareness activities on the Nipah virus have been implemented by government and non-governmental actors. However, community-based evidence suggests a persistent knowledge–behavior gap: in a survey of 545 respondents, only 29.2% had good knowledge and 33.0% reported correct preventive practices, while 53.2% still consumed raw date palm sap, leaving spillover risk largely unchanged [11].

### 3.2 Clinical uncertainty and diagnostic delays

Early clinical presentation of Nipah virus is non-specific, complicating timely recognition. Bangladesh has made substantial progress in expanding laboratory capacity for Nipah virus detection, including improvements in RT-PCR availability and national reference laboratory systems [1]. However, despite these advances, diagnostic confirmation remains largely centralized, and clinical decision-making at peripheral facilities still depends on referral pathways, which can delay escalation of suspected cases.

Additional delays may also occur between hospital presentation, sample collection, transport, and laboratory confirmation. Quantitative data from recent outbreaks in Bangladesh (2023–2025) indicate that the median time from symptom onset to hospital admission was 5 days (range: 2–9 days), while the median time from symptom onset to laboratory confirmation was 6 days (range: 3–13 days) [12]. This suggests that patients often remain under clinical evaluation prior to diagnostic confirmation, reflecting the challenges of recognizing the Nipah virus based on its non-specific early presentation. Importantly, the same study found that fatal cases had a shorter median detection lag (5 days) than survivors (9 days), underscoring that even brief delays in recognition may coincide with rapid clinical deterioration [12].

This variability in clinical outcomes is further complicated by the virological characteristics of Nipah virus infection. The incubation period of Nipah virus typically ranges from 3 to 14 days but may extend up to 45 days [4], complicating early detection and timely suspicion. Together, these factors create a critical gap between clinical suspicion and public health action, where early cases may not trigger alerts unless laboratory confirmation is obtained, potentially delaying response during the most actionable phase of transmission. For high-fatality zoonoses, it is essential to treat probable or suspected cases as meaningful early-warning signals. Otherwise, alerts are triggered only after transmission chains are established.

### 3.3 Fragmented surveillance across sectors

Despite the zoonotic nature of the Nipah virus, surveillance in Bangladesh remains siloed. Progress has been made under the One Health framework, with collaborative investigations between human and animal health sectors [13]. However, these efforts are largely event-driven and lack institutionalization. Routine integration of multi-sectoral data for early risk assessment is minimal, with human health signals rarely linked to animal, wildlife, or environmental information relevant to spillover risk [14].

This gap is particularly evident in the surveillance of reservoir hosts such as *Pteropus* bats. Monitoring of these species remains largely research-driven rather than being embedded within a routine, nationally coordinated early warning system [7]. Existing efforts rely on periodic ecological and virological studies, while biological and logistical constraints—including low viral detection rates, temporal variability in shedding, and the wide mobility of bat populations—further complicate systematic monitoring [7]. Consequently, data generated from bat studies are seldom incorporated into real-time risk assessment frameworks.

The core challenge is not data scarcity but the absence of operational mechanisms to synthesize and integrate signals across sectors in a timely manner. Strengthening early warning therefore requires institutionalized mechanisms for joint risk interpretation, shared situational awareness, and coordinated escalation, instead of building entirely new surveillance infrastructures.

## 4. Why conventional surveillance falls short

Conventional surveillance systems are designed to detect increases in incidence, assuming that rising numbers of cases precede response [15]. This logic fails for the Nipah virus, where low incidence does not equate to low risk. Surveillance thresholds based on confirmed cases delay action and undermine preparedness [16]. In Bangladesh, surveillance systems operate under substantial strain from endemic and emerging health challenges, compounded by resource limitations across many districts and upazilas (sub-districts) [17]. Consequently, rare but high-fatality threats like the Nipah virus are particularly vulnerable to deprioritization when early warning is narrowly defined by case accumulation [8].

Syndromic and event-based surveillance systems have strengthened the detection of Nipah virus over time through coordinated reporting and outbreak investigation frameworks. Longitudinal evidence demonstrates substantial progress in case identification, laboratory confirmation, and inter-agency response capacity between 2006 and 2021 [1]. These gains reflect the expansion of structured zoonotic surveillance platforms, which have improved the country’s ability to detect, confirm, and respond to Nipah virus outbreaks.

Nevertheless, evaluations of surveillance performance indicate that such systems remain fundamentally reactive, as they depend on the presentation of clinically apparent cases to healthcare facilities before signals are generated [2]. Empirical analyses indicate that hospital-based surveillance is inherently incomplete at the district and upazila levels: during 2007–2014, an estimated 119 Nipah virus outbreaks occurred in Bangladesh, of which only 62 (52%) were detected, reflecting substantial under-ascertainment driven in part by variability in healthcare-seeking behavior [8]. More recent community-based findings further support continued heterogeneity in care-seeking patterns for Nipah-like illness, with 68% not presenting to surveillance hospitals and 24% initially seeking informal care, reinforcing limitations in facility-based case capture [9]. This limitation is consistent with broader assessments of infectious disease early warning systems, which highlight reduced effectiveness in detecting low-incidence, high-fatality events prior to their clinical recognition [16].

In parallel, ecological and virological studies indicate that spillover risk may emerge prior to detectable human cases within surveillance systems, particularly in bat–human interfaces shaped by environmental and behavioral factors [7]. Consequently, while current systems are effective for outbreak confirmation and response, their capacity for early warning remains constrained in the context of Nipah virus epidemiology.

## 5. What must change next

To transform early warning for the Nipah virus from a reactive mechanism to a proactive system, several strategic shifts are required. Key proposed shifts include moving from early outbreak detection to risk anticipation, operationalizing One Health through routine cross-sectoral coordination, establishing explicit alert thresholds that permit early action under uncertainty, and linking warning signals to predefined response measures. Together, these proposed shifts are tailored to the epidemiological and health system realities of Bangladesh (Fig 1).

### 5.1 From detection to anticipation

Early warning should incorporate predictive elements grounded in historical patterns, seasonality, and ecological risk. Periods associated with known drivers of spillover should trigger heightened vigilance and preparedness before human cases are detected [18]. In Bangladesh, Nipah virus spillover shows strong seasonal clustering during the winter months (December–March), and winter temperature alone explains 53% of the annual variation in spillover events, supporting a shift from passive detection to anticipatory preparedness [6,18]. Spillover is also not evenly distributed in time or space: temperature explains about 36% of year-to-year variation, while distance to surveillance hospitals explains about 45% of spatial variation, indicating that both ecological and access-related factors shape observed risk [18].

Operationally, a period of heightened alert could be defined annually during this high-risk season, particularly in districts with prior outbreaks. During this period, preparedness measures at the upazila level should be proactively strengthened, including (i) reinforcement of community risk communication on safe consumption practices, (ii) readiness of isolation facilities in Upazila Health Complexes (UHCs), and (iii) pre-positioning of sample collection and transport systems. Such seasonal activation may enable early warning systems to function as time-bound preparedness mechanisms rather than solely case-based response tools.

### 5.2 Operationalizing One Health

One Health should move beyond policy rhetoric to operational integration across sectors. This requires formalized and routine data-sharing mechanisms between human health authorities, such as the Institute of Epidemiology, Disease Control and Research (IEDCR), research institutions such as the International Centre for Diarrhoeal Disease Research, Bangladesh (icddr,b), and sectors responsible for livestock, wildlife, and environmental monitoring [19].

Currently, surveillance of *Pteropus* bats in Bangladesh remains largely fragmented and research-driven rather than systematically incorporated into national surveillance systems [7]. A feasible step toward operationalization is the establishment of structured joint risk assessment processes at the district level. These would be coordinated through Civil Surgeon offices, where human clinical signals, ecological observations such as bat roost proximity, and behavioral risk indicators including date palm sap consumption practices are periodically reviewed. These assessments could be triggered during the defined high-risk season or upon detection of suspected encephalitis cases at the upazila level, with Civil Surgeons responsible for convening cross-sectoral discussions and notifying national authorities where elevated risk is identified. Such integration would support earlier identification of spillover risk prior to laboratory confirmation [19].

### 5.3 Redefining alert thresholds

Alert criteria should be explicitly defined in operational terms and should account for uncertainty. In the Bangladesh context, early warning triggers at the upazila level can be operationalized as the occurrence of at least one suspected Nipah case presenting with encephalitis and a relevant epidemiological exposure, such as the consumption of raw date palm sap [1], or the identification of two or more unexplained encephalitis cases within a seven-day period in the same locality [5]. Additionally, a single probable case occurring during the high-risk season in a previously affected district could prompt early action [2].

Once such thresholds are reached, Upazila Health and Family Planning Officers (UHFPOs) could initiate preliminary response measures, including case isolation, risk communication, and rapid reporting to national authorities, without waiting for laboratory confirmation from IEDCR [5]. This approach requires acceptance of a limited number of false alarms as a necessary trade-off, given the high case fatality and the consequences of a delayed response in Nipah virus outbreaks [20]. However, this trade-off must also be understood in the context of resource prioritization constraints within the health system, including limited workforce capacity, diagnostic resources, and outbreak response funding—particularly in settings where multiple competing health priorities may require simultaneous attention. Such a trade-off does not negate the value of Bangladesh’s existing surveillance system, which has contributed to outbreak detection and containment in past human-to-human transmission events; rather, it highlights that increasing surveillance sensitivity must be carefully balanced against the risk of alert fatigue, unintended stigma for affected communities, and the diversion of these limited public health resources away from competing health priorities.

### 5.4 Linking warning to action

Early warning is meaningful only if it triggers predefined and time-bound measures of preparedness. Clear response protocols—covering infection control, diagnostics, and risk communication—should be activated automatically once predefined alert thresholds are met [3]. In Bangladesh, this requires linking risk signals to existing institutional structures across the health system. At the sub-national level, UHCs and district health authorities should be positioned to initiate initial response measures, while at the national level, IEDCR provides laboratory confirmation and technical guidance. In parallel, community awareness platforms led by the government and non-governmental organizations (NGOs), including icddr,b, represent an important preparedness asset that can be rapidly mobilized for risk communication and community engagement [11].

Strengthening these linkages is important to ensure that early-warning signals translate into timely, coordinated action. However, implementation of such a system should also acknowledge existing constraints, including limited diagnostic capacity outside central laboratories, resource limitations for rapid deployment, and the absence of fully codified trigger-based response protocols.

## 6. Translating early warning into actionable preparedness

Early warning systems are effective only when signals are rapidly translated into timely preparedness actions. For the Nipah virus in Bangladesh, this requires an operational pathway that links risk detection to immediate preparedness measures across the health system hierarchy, ensuring minimal delay between signal recognition and intervention [16,3].

When predefined early warning thresholds (as outlined in Section 5.3) are reached at the upazila level, response actions should be initiated without awaiting laboratory confirmation [14,3]. At the frontline level, UHFPOs, together with designated clinicians at UHCs, should implement immediate patient isolation, initiate preliminary case investigations, and notify district surveillance authorities [2,14]. Simultaneously, rapid community risk communication should be activated to reduce exposure risk, particularly related to raw date palm sap consumption practices. Such communication must be behavior-focused rather than awareness-based, as studies have shown a substantial gap between high reported positive attitudes (94.1%) and much lower adoption of preventive practices (33.0%) [11].

At the district level, the Civil Surgeon’s office could assume coordination responsibility for outbreak verification and response mobilization. This includes the deployment of rapid response teams (RRTs), supervision of infection prevention and control (IPC) measures in health facilities, and ensuring timely transport of clinical and environmental samples to the IEDCR for confirmatory testing [2,5].

At the national level, IEDCR plays a central role in laboratory confirmation, epidemiological investigation, and provision of technical guidance for containment measures [1]. Coordination at this level is also required to ensure standardized reporting, situational updates, and strategic decision-making during outbreak escalation. In addition, collaboration with national and international research and public health partners can further strengthen field epidemiology, advanced data analysis, and operational research during outbreak response [1].

The effectiveness of this system depends critically on preparedness capacity at the upazila level. Health workers in UHCs should be trained not only to recognize the clinical warning signs of Nipah virus infection but also to understand the operational meaning of early warning triggers and their associated response protocols [14]. Strengthening routine reporting systems and ensuring rapid communication channels between upazila and district levels are essential components of this preparedness structure. Evidence from previous Nipah virus outbreaks in Bangladesh indicates that delays in recognition and response significantly contribute to high mortality and onward transmission [8,18]. These findings highlight the importance of integrating clinical, behavioral, and surveillance-based signals into a unified response framework.

Cross-sectoral coordination is equally essential. Collaboration between human health services, veterinary authorities, and wildlife or ecological monitoring systems strengthens the ability to identify and respond to spillover risks in a timely manner [19], particularly given the established role of fruit bats (*Pteropus* spp.) as reservoir hosts [7]. However, operational implementation remains constrained by limited diagnostic capacity outside central laboratories, uneven distribution of trained RRTs, and the absence of fully institutionalized trigger-based activation protocols at sub-national levels [14].

Despite these challenges, linking early warning signals to structured, pre-assigned response actions can substantially reduce the delay between risk detection and intervention. For high-fatality zoonotic diseases such as Nipah virus, even modest reductions in response time can significantly improve outbreak containment outcomes.

## 7. Conclusion

Early warning for Nipah virus in Bangladesh remains constrained not by the absence of surveillance, but by how it is conceptualized and operationalized. Reframing the concept as a process of proactive risk assessment—integrating contextual, clinical, and cross-sectoral signals—offers a more appropriate foundation for preparedness than approaches reliant on laboratory confirmation and case accumulation alone (Fig 1). Strengthening such systems requires accepting uncertainty, redefining alert thresholds, and linking early signals to timely action, while avoiding unnecessary alert fatigue and inefficient use of limited resources. Importantly, this approach does not seek to predict spillover events but to enable structured preparedness in the face of uncertainty. Future efforts should focus on operationalizing and evaluating such frameworks within routine surveillance systems to strengthen preparedness for the Nipah virus and other emerging zoonotic threats.

### 7.1 Scope of this perspective

This article is based on a synthesis of published literature and documented surveillance experiences in Bangladesh and comparable settings. It is intended to provide a conceptual framework for strengthening early warning systems for Nipah virus, rather than prescriptive operational guidance. The perspectives presented should therefore be interpreted in conjunction with context-specific public health, epidemiological, and veterinary expertise.
